# Motives for non-adherence to colonoscopy advice after a positive colorectal cancer screening test result: a qualitative study

**DOI:** 10.1080/02813432.2020.1844391

**Published:** 2020-11-13

**Authors:** Lucinda Bertels, Peter Lucassen, Kristel van Asselt, Evelien Dekker, Henk van Weert, Bart Knottnerus

**Affiliations:** a Department of General Practice, Cancer Center Amsterdam, Amsterdam Public Health Research Institute, Amsterdam UMC, University of Amsterdam, Amsterdam, The Netherlands; b Department of Socio-Medical Sciences, Erasmus School of Health Policy & Management, Erasmus University Rotterdam, Rotterdam, The Netherlands;; c Department of Primary and Community Care, Radboud University Medical Centre, Radboud Institute for Health Sciences, Nijmegen, The Netherlands; d Department of Gastroenterology and Hepatology, Cancer Center Amsterdam, Amsterdam UMC, University of Amsterdam, Amsterdam, The Netherlands; e Netherlands Institute for Health Services Research (Nivel), Utrecht, The Netherlands

**Keywords:** Colorectal cancer, screening, adherence, personalised medicine, qualitative research, the Netherlands

## Abstract

**Setting:**

Participants with a positive faecal immunochemical test (FIT) in screening programs for colorectal cancer (CRC) have a high risk for colorectal cancer and advanced adenomas. They are therefore recommended follow-up by colonoscopy. However, more than ten percent of positively screened persons do not adhere to this advice.

**Objective:**

To investigate FIT-positive individuals’ motives for non-adherence to colonoscopy advice in the Dutch CRC screening program.

**Subjects:**

Non-adherent FIT-positive participants of the Dutch CRC screening program.

**Design:**

We conducted semi structured in-depth interviews with 17 persons who did not undergo colonoscopy within 6 months after a positive FIT. Interviews were undertaken face-to-face and data were analysed thematically with open coding and constant comparison.

**Results:**

All participants had multifactorial motives for non-adherence. A preference for more personalised care was described with the following themes: aversion against the design of the screening program, expectations of personalised care, emotions associated with experiences of impersonal care and a desire for counselling where options other than colonoscopy could be discussed. Furthermore, intrinsic motives were: having a perception of low risk for CRC (described by all participants), aversion and fear of colonoscopy, distrust, reluctant attitude to the treatment of cancer and cancer fatalism. Extrinsic motives were: having other health issues or priorities, practical barriers, advice from a general practitioner (GP) and financial reasons.

**Conclusion:**

Personalised screening counselling might have helped to improve the interviewees’ experiences with the screening program as well as their knowledge on CRC and CRC screening. Future studies should explore whether personalised screening counselling also has potential to increase adherence rates.

**Key points**
- Participants with a positive FIT in two-step colorectal cancer (CRC) screening programs are at high risk for colorectal cancer and advanced adenomas. Non-adherence after an unfavourable screening result happens in all CRC programs worldwide with the consequence that many of the participants do not undergo colonoscopy for the definitive assessment of the presence of colorectal cancer. Little qualitative research has been done to study the reasons why individuals participate in the first step of the screening but not in the second step. We found a preference for more personalised care, which was not reported in previous literature on this subject. Furthermore, intrinsic factors, such as a low risk perception and distrust, and extrinsic factors, such as the presence of other health issues and GP advice, may also play a role in non-adherence. A person-centred approach in the form of a screening counselling session may be beneficial for this group of CRC screening participants.

## Introduction

As colorectal cancer (CRC) is the third most common cancer worldwide, screening is recommended to reduce both incidence of CRC and CRC-related mortality and morbidity [1,2]. This goal is to be achieved by detecting CRC before it becomes symptomatic, or by detecting its relevant precursors, advanced adenomas (AA) [3]. CRC screening has been implemented worldwide, the faecal immunochemical test (FIT) being the first test for detecting faecal occult blood in two-step screening [2]. After a positive FIT result, the advice is to follow up with a diagnostic colonoscopy. The risk associated with a positive FIT result ranges from 2.9–7.8% for CRC and 33.9–54% for AA [4]. The positive predictive value of faecal blood in asymptomatic individuals is higher than that of other risk prediction indicators combined, making a positive FIT result the strongest warning signal for CRC [5–7]. CRCs detected by screening are usually in an earlier stage and have a better prognosis than CRCs detected through symptoms [8]. However, not everyone with a positive FIT adheres to programmatic follow-up by colonoscopy [3,9–11]. The Dutch CRC screening program (Box 1), which has one of the highest participation rates in the world (73% in 2017) has an unexplained non-adherence rate between 11 and 14%. Non-adherence rates to colonoscopy in CRC screening programs range from 8% in Italy to 47% in the United States [2,12]. As over half of FIT-positive (FIT+) individuals have advanced neoplasia, even low non-adherence rates deserve our attention.

Box 1. Characteristics of the Dutch national CRC screening program Characteristics of the Dutch national CRC screening program

- Starting 2014, persons aged 55–75 receive a biennial invitation via postal mail to perform a faecal immunochemical test (FIT)
- An accompanying information leaflet advises persons with symptoms that may indicate colorectal cancer to consult their general practitioner (GP)
- Each participant receives the FIT result by a letter through postal mail
- FIT-positive results are accompanied by a ready-made appointment for colonoscopy consultation within 2 weeks
- FIT-positive patients are requested to obtain a copy of relevant medical history and a medication overview at their GP.
- Follow-up colonoscopy takes place at accredited colonoscopy centre <40km (25 miles) of participants’ home
- Positive predictive value of FIT (cut-off 47 μg Hb/g faeces) was 7.1% for CRC and 39.3% for AA in 2017
- Every Dutch citizen is registered at a GP’s practice
- Each citizen has a mandatory health insurance
- The follow-up colonoscopy is paid by the insurance, with a deductible minimum of 385 euro (2017)

Most previous studies on individuals that do not undergo a colonoscopy after positive screening used quantitative methods such as questionnaires [13] and database research [14–18]. These studies suggested several factors that might be associated with non-adherence. Certain socio-demographic factors are negatively associated with adherence, such as belonging to a minority ethnic group, being in a low socio-economic position or living in a remote area [16,17]. Lifestyle and healthcare factors that negatively influence colonoscopy adherence are poor health behaviour in general, severe disability, high comorbidity burden, high age, and poor access to health insurance [14,16,17]. Other suggested reasons are unwillingness, practical barriers and the conviction that the screening test was falsely positive [15]. We found only two studies of a qualitative nature. A Canadian qualitative study found the following reasons: believing the screening result to be false-positive, fear of colonoscopy, having other health issues and difficulties in communication about the positive result or the colonoscopy appointment [19]. A Danish study found practical barriers, discomfort during the examination, personal integrity, multimorbidity, feeling healthy, not having the energy, a belief that cancer is not present, risk of complications and distrust in the screening test as main themes [20]. Little is known about motives for non-adherence to colonoscopy advice after a FIT + result in the Dutch screening setting, which is different from other European CRC screening settings mainly because it is characterised by ready-made appointments for colonoscopy consultation in case of a FIT + result (see Box 1). In this study, we therefore investigated the motives for non-adherence to colonoscopy advice after a positive FIT result in the Dutch CRC screening program.

## Methods

### Data collection

At the time of our data collection (December 2016 to November 2017), the national screening organisation (RIVM) had not yet made their database with participants’ information available for researchers. Since Dutch GPs still received standard notifications of their patients’ positive FIT results (until December 2017) [21], we tried to recruit participants in GP practices. However, most practices did not register their FIT-positive patients as a separate category. One attempt was made to search for these patients in the database of a GP practice, but this turned out to be time-consuming and only yielded one interview. Therefore, instead, we asked GPs willing to cooperate (*N* = 7) to be alert on the inclusion criteria in their patients. In addition, we recruited participants using an online newsletter from a national elderly organization (ANBO) and on social media (Facebook, Instagram) targeting individuals aged 55–75. The online advertisements offered a link to the study website that offered information about participation, information about the background of the study and the person conducting the interviews (LB), and a contact form for application [22].

An interview guide was developed, which was pilot tested with three participants in the CRC screening program. Pilot interviews were not included in this study. The pilot interviews showed that asking the question ‘what was the reason you did (not) undergo the colonoscopy?’ was ineffective, as reasons were multifactorial. Respondents would start in the middle of their narrative while initially leaving out relevant circumstances. Thus, the interview schedule was adapted, leaving this focussed question out. Instead, respondents were invited to tell their story about their participation in the screening program from beginning to end, while the interviewer would ask ‘why’ and ‘how’ questions where appropriate. Subsequently, we used a semi-structured interview guide (Box S1) based on literature to complement the participant narratives with exploration of themes that were potentially related to their non-adherence [14–17,23–31]. Questions were formulated in an open manner and leading questions were avoided.

Under supervision of BK (PhD, GP), the interviews were conducted in person by LB (MSc. in medical anthropology and sociology, female, PhD candidate) at the participants’ homes. Only the participant and the researcher were present, except for three cases in which the partner of the participant was also present. LB received interview training during her previous education and had experience with carrying out in-depth interviews. She had no prior connection to the topic of cancer screening. Written informed consent was obtained prior to the interviews. The interviews lasted between twenty minutes and two hours, with an average of 40 min. The medical ethics committee of the Amsterdam UMC (location AMC) granted a waiver for this study.

### Participants

The main researcher (LB) would phone applicants to schedule an interview if inclusion criteria were met. Inclusion criteria were: a positive FIT result in the past two years and not having undergone a colonoscopy within 6 months after the FIT result. Participants were offered a 20-euro gift certificate. As participants were part of a hard-to-reach population [32], we included all participants who met the inclusion criteria until data saturation was reached. We excluded participants who had undergone a colonoscopy within three years prior to the FIT + result, as this is seen as a contra-indication for colonoscopy by the Dutch screening organisation [33].

**Figure 1. Flowchart of recruitment and inclusion.**
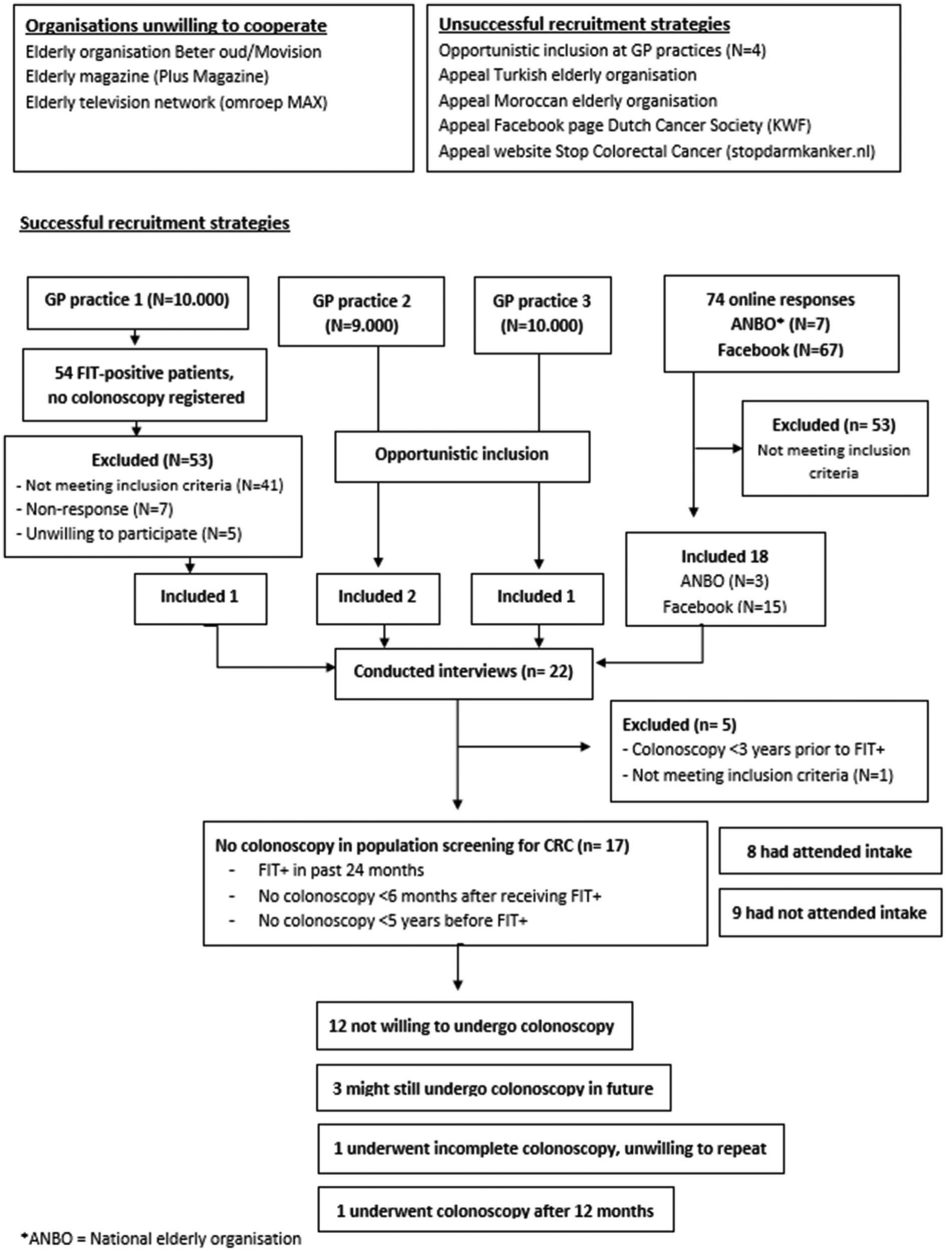


Twenty-two persons were interviewed (Figure 1). One participant did not meet the inclusion criteria and was thus excluded. Four other participants had undergone one or more colonoscopies within three years before the positive FIT result. As a recent colonoscopy is a medical exclusion criterion not to undergo another screening colonoscopy, we also excluded these four participants from the analysis. Thus, 17 participants were included in this study (see Table 1).

**Table 1.** Participant characteristics.

Characteristics	Participants
Number of participants	17
Age range participants, years	
55–64	9 (53%)
65–74	8 (47%)
Sex	
Female	11 (65%)
Male	6 (35%)
Recruitment method	
Elderly organization	2 (14%)
Social media	11 (67%)
General practice	4 (19%)
Time between FIT + and interview	
Range, months	2−18
Median, months	10,5
Educational level	
Low	5 (29%)
Middle	6 (38%)
High	6 (33%)
Financial strain	
No financial strain	11 (67%)
Minor or major financial strain	6 (24%)
No answer	1 (5%)
Ethnicity	
Western-European	16 (95%)
Other	1 (5%)

### Analysis

The interviews were audio-recorded, transcribed, anonymized and checked for accuracy. Field notes taken during the interviews were added to the transcripts – these were notes on respondents’ emotional responses, intonation, and medical history or appointment letters by the screening organisation that proved helpful in detailing their narrative. Data collection and thematic analysis of data were done simultaneously; LB, BK and KvA discussed the content of the interviews and the level of data saturation after every five to seven interviews. The discussions after the first few interviews led to adaptations of the interview guide. We reached data saturation after the 17 interviews included in this study, which we defined as the point where no new main themes were found in three consecutive interviews (interviews 15–17) [34,35].

LB coded all interviews with open coding using MXQDA software [35]. Another researcher (PL; GP, researcher, experienced in qualitative research) also coded eight interviews and discussed with LB how the codes could be divided into themes. The research team (LB, PL, BK and KA) discussed the code system and the themes until agreement was reached. A native English speaker from a translation agency translated the quotes. The study was reported in accordance with the 32-item checklist of Consolidated Criteria for Reporting Qualitative Research (COREQ) (Table S2).

## Results

### Reasons for non-adherence to colonoscopy advice

The 17 participants mentioned different reasons for non-adherence. They all described a unique narrative with an interplay of multiple themes. These themes were categorized into three key themes: a preference for more personalised care, intrinsic motivations and extrinsic motivations. These themes are explored below.

### Preference for more personalised care

#### Aversion against design of screening program

A number of participants described aversion against the way the screening program was organized, finding it too directive. They mentioned that they did not like the pre-planned appointment for a colonoscopy consultation. They described this as unfriendly, rigid or imposed.

They had already made an appointment for me; they do not call you to ask if the day and time are okay. So I thought to myself "no". This should happen by mutual agreement. (R02)

I thought this was odd, that they had arranged this without asking me which hospital I would have preferred. I did found out I could change the appointment to another hospital, but that was not the important thing: It was more the tone of the letter that got to me. It was so compelling; I do not like that at all. (R13)

### Distrust

A few participants described how this led to feelings of distrust towards either the colonoscopy clinic, the hospital, or the screening organization. Some questioned these institutions’ financial stake in the screening program. A disbelief in the outcome of the FIT was also mentioned; one participant had doubts about blood being present in the stool, another had doubts about the correct interpretation of the test. One participant thus described a phone call to the screening organization, detailing distrust towards both the screening organization and the hospital:

Yes, I cancelled it. But then the lady started really pushing. I say: ‘It is a hospital I do not know at all. I live somewhere else. I get the address of a hospital I know nothing about.’ I thought that was odd. Is that what is behind it? What is your deductible excess? I do not know at all. And I think they were somewhat beating around the bush when it came to those things. (R17)

### Expectations of personalised care during consultation for colonoscopy

Some participants were surprised by the content of the consultation when they discussed the appropriateness of the colonoscopy. They had expected an opportunity to discuss the positive FIT and what it meant to them. However, they experienced it as a medical consultation for the preparation for the colonoscopy, and felt there was little room for discussion:

I wanted that appointment with the consultation nurse because I wanted to know what the alternatives were for the colonoscopy, and consequences of not partaking. But the nurse just told me I should not have come to the appointment if I did not plan to have the colonoscopy to begin with. (R05)

I did not have the opportunity to discuss my fear and reluctance to do the investigation. The nurse was just writing down my medical history. (R04)

### Emotions associated with experiences of impersonal care

Some participants detailed how their experience with the screening program felt impersonal, and how it made them feel pressured into undergoing the colonoscopy. This was sometimes associated with strong emotions. One participant had to excuse himself for a few minutes because emotions overwhelmed him. We heard accounts of feeling stripped of self-determination, loss of control, feeling vulnerable and feeling left at the mercy of physicians when talking about how they experienced their participation in the screening programme.

The moment you go there to go do the colonoscopy, you are at their mercy. You have to subject to what they do and what they decide. (R04)

I really disagreed with the way things were going. It made me feel like I had lost control of my own life. […] You are taken out of the equation as a human being, I think. (R06)

For one participant this led to feelings of resistance and anger:

They are trying to persuade you. I am sure it is well intentioned, they want to help you, but… if you see that someone is terrified, do not keep nagging me like that. I only get angry. (R07)

### Alternative strategies to colonoscopy

Many participants wished to be examined by other means than the colonoscopy that was offered. Firstly, we heard strategies to detect colorectal cancer if it was present, such as a second FIT. This is not something the screening organization was willing to supply, which for some participants was cause for frustration and feeling unheard.

I just wanted to do a second test, because I felt that the Ibuprofen I was taking at the time had caused the test to be unfavourable. I could not get a second opinion. ‘But I am willing to pay for it!’ I said. No. They would not let me have one. (R06)

A few others had this second FIT done with their GP, with a negative outcome that reassured them. Some mentioned that a second positive FIT would make them reconsider colonoscopy: ‘If I get it next time and it is another unfavourable result I think that I probably would do it.’ (R09) Furthermore, we heard the wish for a colonoscopy under general anaesthesia, a CT colonography, the use of a dog trained in detecting cancer, a blood test or a laboratory test on the blood already found in the stool.

Secondly, the participants described strategies to catch CRC in time. One strategy mentioned was to pay attention to blood in the stool and physical complaints such as stomach aches and a change in stool pattern. If such complaints would arise, participants described they would then visit a doctor and consider a colonoscopy.

I do pay more attention with toilet visits. But it is all still the same and I have not seen any blood. The smell is also the same… […] I would definitely go to the doctor if I noticed something. I would not risk it. (R11)

A few participants described getting regular check-ups at their GP to ensure good health as another way to catch CRC in time:

In the last few years, I have been going regularly and he checks my blood pressure every year, and I have had blood tests done several times a year. He thinks that I am healthy. (R10)

### Desire for personalised screening counselling

Several participants described how personal screening counselling might have made a difference in how they experienced their participation in the screening program. They mentioned the desire to have a personal conversation with someone to discuss the FIT outcome and its’ consequences.

For the screening organisation, they say if I do not follow their advice then I am a care refuser. I find that so cold. I would rather just have an appointment to talk things over. (R06)

If it would have gone another way then maybe the nurse could have persuaded me to do it. Now, I just thought they want as many patients as possible because it makes them money. (R04)

Furthermore, they had a wish to discuss their ideas about alternatives for the colonoscopy. This is something they felt they had lacked.

I did want that appointment with the consultation nurse; in case she could give me advice on what to do other than the colonoscopy. (R05)

### Intrinsic motivations

#### Perception of low risk for CRC

In conjunction with the above described preference with more personalised care than the CRC screening program could offer, we heard all participants describe a low perception of risk for CRC. This was demonstrated by a lack of worry about CRC or a low assessment of their risk of developing CRC. Most participants described both.

I think my risk is less than other peoples’ are. (R08)

Only in a very small percentage of people does it really turn out to be something. I am not one of those people. I just know that. (R09)

When asked to explain, we often heard alternative explanations for the positive FIT. Some mentioned that they thought that the blood in their stools could be a one-time coincidence and thus did not hold any meaning. Other explanations were haemorrhoids, constipation, stress, a consequence of previous surgery, an anal fissure, or a polyp.

I have haemorrhoids, so if there is blood in my stool that is why. […] If I get another invitation for this test in two years, I will make sure to avoid spicy food beforehand so they will not open up and bleed again. (R02)

Another explanation for having a low risk for CRC was related to bodily sensations. These included not having any symptoms of CRC, feeling healthy, not looking sick and knowing the body.

I was not worried. Nothing else was bothering me. No stomach pain or this or that, so I thought: never mind. (R15)

A third, less frequently mentioned explanation was living a healthy life. Arguments were exercising, eating healthy, and not smoking or drinking excessively. A final argument was having a negative family history for CRC. One participant alluded to this notion as his family commonly had colorectal polyps instead of cancer.

### Aversion to, and fear of, colonoscopy

Participants commonly reported an aversion to having a colonoscopy. Mainly, apprehension for the colonoscopy preparations such as a change in diet, fasting, drinking the colonoscopy preparation liquid, and not being able to take anticoagulants. We also heard expectations of pain and embarrassment. The feelings of fear and aversion stemmed from previous experiences, experiences of relatives or from information found online or in a flyer.

They told me the colonoscopy would take place under sedation. I told them ‘no way’. That did not help eight years ago when I had it, and I decided then that I would never do this again. (R14)

A less frequently mentioned theme was having fear of the colonoscopy as a medical procedure. These were described as feelings of anxiety raised by the idea of the colonoscope entering the body or the removal of bodily tissue such as polyps: ‘I do not panic easily. But at the thought of that tube going into me… I panic.’ (R07) Worry about the possibility of colonoscopy complications was also mentioned, such as the worsening or return of irritable bowel syndrome symptoms or a fistula.

### Comparison with breast cancer screening

Three women mentioned they took a positive FIT result less serious than a positive mammography in breast cancer screening. One participant did not know why she felt that way. Another participant described she did not trust a test that had an unfavourable outcome when the test had only been performed once. With mammography screening, she had had a few favourable screening results and would thus find an unfavourable result more reliable. The last explained she had difficulty with interpreting the FIT result:

Because with a mammogram, you can really see something. There is something visible and they will tell you: ‘We see something’, so I can imagine what that is like. But when they say: ‘There is something, we found blood…’ Okay, how much blood was there? I find that a little vague. (R11)

### Reluctant attitude to treatment of cancer and fatalism

Some participants had a reluctant attitude towards cancer treatments, especially chemotherapy. Other participants described a fatalistic attitude towards the possibility of developing and treating cancer.

If you are meant to die of cancer, you will die of cancer. They remove your uterus, remove your breasts, it does not matter, you will get cancer on a finger, on your pinkie, something like that. (R16)

### Extrinsic motivations

#### Other health issues or priorities

Some participants mentioned they had other things on their mind at the time of the FIT outcome. Some participants had a parent or a close friend with acute health issues to take care of, but they mainly described their own health issues. We heard about acute issues such as kidney stones or shin splints, which distracted participants from thinking about the colonoscopy.

I had these horrible attacks of kidney stones that led to multiple hospital admissions and then the outcome of this test with that… I always have to do one thing at a time. (R12)

Some patients told about recent health issues that needed prolonged treatment, which were described as leading to feelings of mental tiredness with regard to medical interventions. Similar feelings were described by participants who had experienced a critical life event in the previous years, such as a cardiac arrest, brain tumour or the loss of several persons close to them. These events had had such an impact on their lives that the outcome of the FIT seemed less important in comparison: ‘It is just that I have gone through so much that I see this actually as… Not important is not the right word, but less.’ (R06)

### Practical barriers

Some participants found the time or place of the ready-made appointment for colonoscopy consultation or the colonoscopy itself inconvenient because it was difficult to combine with their work, due to transport strain, travel distance or unfamiliarity with the location.

‘I would have to go all the way to that specific hospital and I do not have a car. Also, there were road works at that time, so a large part of the route was blocked, too. (R03)

### The influence of the GP

A minority of the participants reported that their GP had played a role in their decision not to undergo a colonoscopy. Firstly, the GP sometimes offered either an alternative examination in the form of a second FIT or a blood test or an alternative explanation for the positive FIT. One participant recounted an appointment with the GP a few weeks after receiving the positive FIT result, in which the GP seemed to discourage the colonoscopy:

I went to my doctor and talked about it with him and he sort of put my mind at rest, and said: “If you had come to me with that problem I would not have sent you for a colonoscopy or other tests.” (R10)

Secondly, we heard an example of a GP using a conversation style that the participant did not like. This participant felt disregarded because when his GP contacted him to discuss the positive FIT, he called him by telephone instead of having a face-to-face conversation, speaking in what he felt was an indifferent tone. It seemed that not the message itself, but the way it was brought led this participant to not undergo the colonoscopy. Alternatively, some participants described that their current or previous GPs had an encouraging attitude towards colonoscopy after blood was found to be present in the stool (either by FIT or participants’ visual inspection).

### Financial reasons

In the Dutch health insurance system, every citizen has a yearly obligatory deductible excess that can be raised by a voluntary deductible excess in exchange for lower monthly health insurance contribution (see Box S3). Two participants described that their heightened deductible excess was part of the reason for non-adherence.

Just that year I had raised my deductible to 875 euro, so I really was not eager to do the examination. (R01)

All other participants said their deductible excess did not play a role. Some were unaware that a colonoscopy would have to be partly paid for through their deductible excess; others had already paid their deductible in full.

## Discussion

### Summary

The participants in this study described several motives for non-adherence to colonoscopy advice after a positive FIT result in the Dutch CRC screening program. We divided these into a preference for more personalised care, intrinsic motives and extrinsic motives. The preference for more personalised care was expressed through aversion against the design of the screening program, expectations of personalised care, emotions associated with experiences of impersonal care and a desire for counselling where options other than colonoscopy could be discussed. As extrinsic motives, we found having other health issues or priorities, practical barriers, a negative or trivializing attitude of the GP and financial reasons. For intrinsic motives, the most dominant theme was a low risk perception for CRC. Explanations for this low risk perception included having an alternative explanation for the occult blood found in the stool, not experiencing symptoms, feeling healthy, living a healthy life and having a negative family history of CRC. Other intrinsic motives were aversion and fear of colonoscopy, distrust towards the colonoscopy clinic, hospital or screening organization, reluctant attitude to treatment of cancer and fatalism.

### Strengths and limitations

After initial difficulties in finding an effective recruitment strategy, social media recruitment eventually proved to be effective. Although not all participants were recruited this way, this strategy could have led to a selection bias of participants who have computer skills and visit social media sites. Another possible consequence of this recruitment strategy could be that we recruited a majority of female participants, as women tended to be more active on Facebook. Possibly, more male participants would have yielded other topics. Furthermore, all but one of the participants were western European, making the sample ethnically homogeneous. This limits our results to this specific group of individuals, and it is possible that non-white participants have different ideas or attitudes that we have not covered in this study. Finally, as a gift card was offered and most of our respondents were recruited by means of self-selection, this might have introduced a self-selection bias where selection was skewed towards individuals under financial strain and individuals feeling a desire to detail their experiences. However, most participants did not report being under financial strain and we heard a wide range of experiences, thus we feel this did not affect our results. The length of the interviews varied, since one interview was very short (20 min) and one interview was very long (two hours). We however do not feel this affected our results as most other interviews lasted approximately 40 min.

### Interpretation of results

One of the main themes we found was a preference for more personalised care. This theme has not been discussed in previous literature on non-follow up in CRC screening. A possible explanation for this new finding might be the unique way the Dutch screening program was organized; with a pre-made appointment for colonoscopy consultation accompanying the FIT positive result letter and rapid colonoscopy hereafter [36]. Furthermore, some of the interviewed participants demonstrated a lack of knowledge of CRC, such as the asymptomatic nature of early stage CRC and the fact that one can suffer from haemorrhoids and CRC simultaneously. Moreover, knowledge on certain aspects of the screening program seemed to be lacking; such as the awareness that after an unfavourable FIT result the next screening round will consist of another invitation for colonoscopy and not another FIT.

### Comparison with existing literature

The intrinsic and extrinsic motives we found in this study were all mentioned in previous literature on the topic of non-follow up in CRC screening [19,20,25,26], and there were no main themes discussed in other recent qualitative studies that we did not find in this current investigation [19,20]. We will therefore focus on the preference for more personalised care.

There is an ongoing discussion about how standardization in healthcare may sometimes clash with current ideas of personalised care [37,38]. The Dutch CRC screening program is characterised by a default invitation and a premade appointment for a medical consultation for colonoscopy after a FIT+ [36]. Both strategies are also used in screening programs for other cancers, such as the breast cancer-screening program in Malta [39]. Such strategies are examples of standardization of care, where guidelines have been designed based on the best available evidence in order to gain the best outcome for the largest number of individuals [37,38,40]. Research has shown that it is effective, as screening decreases mortality of CRC [41–43]. Moreover, it was established that prescheduled appointments have a higher compliance rate than open appointments [44]. In the Dutch setting, the focus of the program is on informed decision-making, as is the case with other cancer screening programs worldwide [39,45,46]. Participants are provided with information and are expected to make an autonomous decision on (further) participation [47]. A health professional is usually not involved in this process [48]. The use of informed decision-making has increased in the past decades [47,49] but research has shown that making an autonomous decision is not always possible or preferred by patients [50]. Furthermore, there are social norms associated with making a ‘good’ decision in an organised screening program [48,51] and it has been documented that these characteristics could also contribute to feelings of guilt if invitees choose not to (further) participate [51]. A focus on shared decision making – where a decision is made in collaboration with a health professional – would perhaps have been a better alternative for some participants in this study. Several studies found that in making CRC screening decisions most individuals prefer to consult their physician [52–54]. This might be especially the case for vulnerable patients such as racial/ethnic minorities and low-income individuals as these groups exhibit lower screening uptake [55]. This shared decision making should not only focus on the colonoscopy as a yes or no option, but should also function as a counselling opportunity to offer participants emotional support and strategies for coping with the positive FIT  in case of not (yet) undergoing the colonoscopy. This may improve FIT positive participants' experiences in the programme. At the same time, a counselling moment could be an opportunity to address any gaps in knowledge and misunderstanding surrounding CRC and CRC screening. As previous studies have shown that improving knowledge may increase uptake and adherence rates in CRC screening, this counselling moment has potential to improve adherence rates as well [56–58].

### Implications for practice

The current design of the screening program does not leave much room for a consultation to discuss participants’ options, while this may be what the interviewed participants could have benefited from. This would most likely have improved their experiences with the screening program, has the potential to increase knowledge levels regarding CRC and CRC screening and may improve the way participants experience decision-making. We suggest exploring the potential for including a moment of shared decision making for CRC participants with a FIT + test who are inclined not to do a follow up colonoscopy. This could take place in the form of a screening counselling session with a focus on exploration of the participant’s ideas and concerns, improving their knowledge, offering emotional support and discussing alternatives for colonoscopy.

## Conclusion

Some of the interviewed participants described a negative experience in the Dutch CRC screening program. FIT positive participants who were not willing to undergo a recommended colonoscopy in the Dutch CRC screening programme may benefit from personalised screening counselling. This could potentially improve their experience with the screening program as well as their knowledge on CRC and CRC screening. As this may also increase adherence rates of colonoscopy after screening, counselling for FIT positive participants who have doubts about undergoing the colonoscopy should be explored in further studies.

## Supplementary Material

Supplemental Material
