# Supplementary material for: Motives for non-adherence to colonoscopy advice after a positive colorectal cancer screening test result: a qualitative study
Source: Scand J Prim Health Care. 2020 Nov 13;38(4):487–98. doi: 10.1080/02813432.2020.1844391 (PMC7781896; doi:10.1080/02813432.2020.1844391)
Supplement: Supplemental Material [file IPRI_A_1844391_SM9075.docx]

**Supplementary data**

**Manuscript:** Non-adherence to colonoscopy advice after a positive colorectal cancer screening test result: a qualitative interview study

**Box S1: Semi-structured interview guide**

*Non-adherence to colonoscopy advice*

1. Please tell me about your participation in the CRC screening program from start to finish.
   1. *Probes:* reasons for participation, reaction to test results, estimation of risk at the time, amount of worry at the time, contact with family, friends and physicians.
   2. All decision making moments in this narrative would be further examined by asking ‘why’ and ‘how’ questions.
2. What did you know about the consequences of an unfavourable test result?
3. What was your attitude towards the colonoscopy?
4. Can you tell me about how the intake went? (*when applicable*)
5. To what extent did you have contact with your GP during at the time of the FIT+?
6. Did you, at the time, know anything about why there was blood found in your stool?
7. What are your thoughts in hindsight about your participation?
8. Could you name anything that would have changed the decision you made about not undergoing the colonoscopy?
9. What are your thoughts about the CRC screening program in general?
10. Compared to the average person your age, what do you think your chances are of developing colorectal cancer now?

**Table S2: Consolidated criteria for reporting qualitative studies (COREQ): 32-item checklist**

Developed from:

Tong A, Sainsbury P, Craig J. Consolidated criteria for reporting qualitative research (COREQ): a 32-item checklist for interviews and focus groups. *International Journal for Quality in Health Care*. 2007. Volume 19, Number 6: pp. 349 – 357

| **No. Item** | **Guide questions/description** | **Reported on Page #** |
| --- | --- | --- |
| **Domain 1: Research team and reﬂexivity** |  |  |
| *Personal Characteristics* |  |  |
| 1. Interviewer/facilitator | Which author/s conducted the interview or focus group? | Page 3 |
| 2. Credentials | What were the researcher’s credentials? E.g. PhD, MD | Page 3 |
| 3. Occupation | What was their occupation at the time of the study? | Page 3 |
| 4. Gender | Was the researcher male or female? | Page 3 |
| 5. Experience and training | What experience or training did the researcher have? | Page 4 |
| *Relationship with participants* |  |  |
| 6. Relationship established | Was a relationship established prior to study commencement? | Page 3 |
| 7. Participant knowledge of the interviewer | What did the participants know about the researcher? e.g. personal goals, reasons for doing the research | Page 3 |
| 8. Interviewer characteristics | What characteristics were reported about the interviewer/facilitator? e.g. Bias, assumptions, reasons and interests in the research topic | Page 4 |

| **Domain 2: study design** |  |  |
| --- | --- | --- |
| *Theoretical framework* |  |  |
| 9. Methodological orientation and Theory | What methodological orientation was stated to underpin the study? e.g. grounded theory, discourse analysis, ethnography, phenomenology, content analysis | Page 4 |
| *Participant selection* |  |  |
| 10. Sampling | How were participants selected? e.g. purposive, convenience, consecutive, snowball | Page 3 |
| 11. Method of approach | How were participants approached? e.g. face-to-face, telephone, mail, email | Page 3 |
| 12. Sample size | How many participants were in the study? | Page 4 |
| 13. Non-participation | How many people refused to participate or dropped out? Reasons? | Page 4 (figure 1) |
| *Setting* |  |  |
| 14. Setting of data collection | Where was the data collected? e.g. home, clinic, workplace | Page 4 |
| 15. Presence of non-participants | Was anyone else present besides the participants and researchers? | Page 4 |
| 16. Description of sample | What are the important characteristics of the sample? e.g. demographic data, date | Page 5 (table 1) |
| *Data collection* |  |  |
| 17. Interview guide | Were questions, prompts, guides provided by the authors? Was it pilot tested? | Additional file and page 4 |
| 18. Repeat interviews | Were repeat inter views carried out? If yes, how many? | Not done |
| 19. Audio/visual recording | Did the research use audio or visual recording to collect the data? | Page 3 |
| 20. Field notes | Were ﬁeld notes made during and/or after the inter view or focus group? | Page 4 |
| 21. Duration | What was the duration of the inter views or focus group? | Page 4 |
| 22. Data saturation | Was data saturation discussed? | Page 4 |
| 23. Transcripts returned | Were transcripts returned to participants for comment and/or correction? | Not done |
| **Domain 3: analysis and ﬁndings** |  |  |
| *Data analysis* |  |  |
| 24. Number of data coders | How many data coders coded the data? | Page 4 |
| 25. Description of the coding tree | Did authors provide a description of the coding tree? | Page 4 and supplement 2 |
| 26. Derivation of themes | Were themes identiﬁed in advance or derived from the data? | Page 4 |
| 27. Software | What software, if applicable, was used to manage the data? | Page 4 |
| 28. Participant checking | Did participants provide feedback on the ﬁndings? | Not done |
| *Reporting* |  |  |
| 29. Quotations presented | Were participant quotations presented to illustrate the themes/ﬁndings? Was each quotation identiﬁed? e.g. participant number | Page 6 to 15 |
| 30. Data and ﬁndings consistent | Was there consistency between the data presented and the ﬁndings? | Yes, there was.  Page 6 to 10 |
| 31. Clarity of major themes | Were major themes clearly presented in the ﬁndings? | Yes, they were.  From page 6 to 10 |
| 32. Clarity of minor themes | Is there a description of diverse cases or discussion of minor themes? | Description of diverse cases in supplement 2, minor themes in page 6 -10 |

**Box S3: Dutch health insurance and deductible excess**

- Basic health insurance is mandatory for every Dutch citizen
- The healthcare insurance is paid with a monthly premium that an individual pays to their healthcare insurance of their choice.
- Individuals pay the first part of their medical expenses themselves. This is called the deductible excess. The insurance company covers the rest of the expenses.
- At the beginning of each year, individuals can choose a deductible excess between 385 and 885 euro. The lower the deductible excess they choose, the higher their monthly premium will be during that year.
- Some types of medical care are covered without deductible excess, such as appointments with the GP, medical care in an emergency room, obstetric care, maternity care, dental care for children under 18 and the first step of population based cancer-screening programmes.
- A FIT is covered without deductible excess, but a colonoscopy is not.
